# Biofilm sensitization to break Staphylococcus aureus tolerance to cold atmospheric plasma therapy

**DOI:** 10.1099/jmm.0.002193

**Published:** 2026-08-20

**Authors:** Abdullah Baz, Jontana Allkja, Muhanna Alshehri, Yao He, Zainab Bilal, Eve Hughes, Olivia Sealy, Craig Williams, Karen Faulds, Gordon Ramage, Jason L. Brown

**Affiliations:** 1 School of Medicine, Dentistry & Nursing, College of Medical, Veterinary and Life Sciences , University of Glasgow, Glasgow, UK; 2 Department of Biological Science, Faculty of Science, King Abdul Aziz University, Jeddah, Saudi Arabia; 3 Research Centre for Health, School of Health and Life Sciences, Glasgow Caledonian University, Glasgow, UK; 4 Department of Chemistry, Open Innovation Hub for Antimicrobial Surfaces, Surface Science Research Centre, School of Physical Sciences, University of Liverpool, Liverpool, UK; 5 Department of Pure and Applied Chemistry, Centre for Molecular Nanometrology, Technology and Innovation Centre, University of Strathclyde, Glasgow, UK; 6 Microbiology Department, Lancaster Royal Infirmary, University of Lancaster, Lancaster, UK

**Keywords:** antibiotics, biofilms, cold atmospheric plasma, H_2_O_2_, KHS101, sensitization, *Staphylococcus aureus*

## Abstract

**Introduction.** Biofilm-associated infections present a major therapeutic challenge due to their intrinsic tolerance to conventional antimicrobials. Cold atmospheric plasma (CAP) has shown promise as a non-antibiotic antimicrobial modality; however, some bacteria including *Staphylococcus aureus* can exhibit tolerance to plasma exposure.

**Gap Statement.** Strategies that sensitize CAP-tolerant biofilms to plasma treatment may improve CAP efficacy, but suitable adjunctive compounds and mechanisms remain poorly defined.

**Aim.** This study aimed to determine whether repurposed bioactive compounds could enhance CAP activity against *S. aureus* biofilms.

**Methodology.** Selected compounds from the Tocriscreen™ bioactive compound library were initially screened, followed by treatment of *S. aureus* biofilms with KHS101 ±CAP therapy. Biofilm viability was quantified using live/dead qPCR. To probe mechanisms of sensitization, biofilms were exposed to H_2_O_2_ at concentrations equivalent to those generated by CAP, either alone, or in combination with KHS101 or conventional antibiotics. Various microscopy techniques were used to visualize the cellular impacts of KHS101, while metabolic activity and cell viability of dual therapies were determined using AlamarBlue^®^ assay and plate count assays, respectively.

**Results.** Short-term KHS101 treatment alone displayed modest antibiofilm activity at concentrations that inhibited planktonic growth. However, pre-treatment with KHS101 followed by CAP therapy resulted in significant reductions in viable populations in *S. aureus*-containing biofilms. Microscopy revealed structural perturbations consistent with cellular stress following KHS101 exposure, but also showed intact cellular ultrastructure. Mechanistic probing demonstrated that equivalent concentrations of H_2_O_2_ with KHS101 were insufficient to reproduce the enhanced efficacy observed with CAP. In contrast, H_2_O_2_ enhanced flucloxacillin activity in a strain-dependent manner, sensitizing *S. aureus* biofilms to otherwise sub-lethal concentrations of antibiotic.

**Conclusion.** These findings demonstrate that tolerance of *S. aureus* biofilms to CAP can be overcome through dual-therapy strategies. Treatment with the repurposed compound KHS101 was associated with enhancement of CAP efficacy via an unknown mechanism. However, the inability of H_2_O_2_ to reproduce this effect highlights the importance of additional plasma-derived reactive species in mediating this dual-action killing. Together, these findings position biofilm sensitization as a central concept emerging from this study, whereby a non-lethal adjunct can lower the threshold for CAP-mediated killing without acting primarily as a direct antimicrobial.

## Data Summary

Raw data supporting the findings of this study are available from the corresponding author upon reasonable request.

## Introduction

Biofilm-associated infections represent a major and persistent clinical challenge, largely due to the intrinsic antimicrobial tolerance conferred by biofilm formation [1]. One bacterial pathogen, *Staphylococcus aureus* is amongst the most problematic biofilm-related protagonists in this context. *S. aureus* is often associated with nosocomial infections arising from resistance to front-line antibiotics such as methicillin: methicillin-resistant *S. aureus* (MRSA) is the leading bacterial cause of death [2]. Compounding this clinical burden, *S. aureus* continually colonizes the nasal passage of up to 20% of the human population, with a further 30% intermittently [3]. More recent studies have suggested that the intestines harbour *S. aureus* in higher quantities than the nasal passage [4]. Ultimately, these niches create a large reservoir of carriers that facilitates transmission within both healthcare and community settings and increases the likelihood of opportunistic infection. Worryingly, carriage of MRSA is also ~5% within the community [5, 6], further compounding the concern.

To date, MRSA resistance to other antibiotics has been reported [7]. *S. aureus* readily acquires this genetic resistance through mutation and horizontal gene transfer, exemplified by the emergence of MRSA via acquisition of the *mec* genes. These genes, such as *mecA,* give rise to mutations in enzymes responsible for crosslinking peptidoglycan chains in the bacterial cell wall: proteins that are otherwise targeted by the β-lactams [8]. A variety of other gene mutations can arise in MRSA (e.g. blaZ, tetK, ermA) giving rise to resistance to other β-lactams as well as other classes of antibiotics [7, 9]. However, genetic resistance alone does not fully explain survival to antibiotics, as *S. aureus* can also evade antimicrobial killing through adoption of the biofilm lifestyle. As with most biofilm forming micro-organisms, the staphylococcal sessile phenotype differs considerably from planktonic cells, encompassing the formation of protective extracellular matrix and altered metabolic states [10]. As a result, conventional antibiotic treatments frequently fail to eradicate biofilm-associated infections, even in ‘susceptible’ communities leading to a ‘tolerant’ and/or ‘persister’ like phenotype characterized by 100–1,000-fold increase in the likelihood of antibiotic survival [11]. Tolerance refers to phenotype-dependent survival without necessarily involving genetic resistance to the treatment, while persister cells represent a surviving subpopulation commonly linked to reduced metabolic activity [12]. These problematic changes within biofilm communities underscore the urgent need for alternative or adjunctive therapeutic strategies.

One particular alternative method involves cold atmospheric plasma (CAP), which has emerged as a promising antimicrobial technology capable of inactivating a broad spectrum of micro-organisms, including antibiotic-resistant bacteria. Several recent investigations have demonstrated promising outcomes with CAP, establishing its effectiveness against planktonic micro-organisms and exploring its anti-biofilm activity [13–16]. CAP generates a complex and dynamic mixture of reactive oxygen and nitrogen species (RONS) that can impose oxidative stress on microbial cells, offering a mode of action distinct from most traditional antimicrobials. This is evidenced by its application as direct therapies, and when delivered in an indirect form, e.g. plasma-activated water [17, 18]. While CAP has shown potent antimicrobial activity in a range of experimental systems, its efficacy against established biofilms is more variable, with evidence reporting that biofilm-embedded bacteria can tolerate plasma exposure under certain conditions [19–21]. This highlights both the potential of CAP as an innovative antimicrobial modality whilst also outlining that we need to understand the factors that limit its effectiveness against some biofilm communities.

To further enhance CAP efficacy and circumvent any tolerant phenotypes, recent studies have investigated the potential of combining plasma with antibiotics, antimicrobial and other novel techniques [14, 22–25]. One potential avenue that is beginning to be explored is assessing the antimicrobial activity of repurposed compounds. To this end, drug repurposing represents an attractive strategy for addressing biofilm-associated infections, as it enables the identification of new antimicrobial applications for compounds with established chemical and safety profiles, which can circumvent lengthy development timelines associated with conventional *de novo* drug discovery [26–28]. In this context, curated compound collections such as the Tocriscreen™ bioactive compound library provide a valuable platform for uncovering potential therapeutic interventions against planktonic and sessile cells. Abduljalil *et al.* [29] recently reported that Tocriscreen™-derived molecules exhibited various antibiofilm activity against several key fungal species, including *Candida albicans*, *Candida auris* and *Nakaseomyces glabrata* [29]. Building on this previous Tocriscreen™ bioactive compound library study, we selected three of the most effective compounds, KHS101, darapladib and polygodial, for evaluation against *S. aureus*. We aim to pair the most effective compound with CAP in the hope of sensitizing *S. aureus* and lowering the threshold for CAP antimicrobial killing. To do this, we primarily focus on chronic wound-associated biofilms because this clinical context is particularly relevant to CAP-based approaches, as plasma can be applied topically and has been explored for the treatment of infected or non-healing wounds [30]. We ultimately hope to investigate the potential use of repurposed compounds in breaking our previously reported *S. aureus* tolerance to CAP therapy.

## Methods

### Microbial growth and standardization

A detailed breakdown of microbial strains, their source and genetic backgrounds are detailed in Table S1, available in the online Supplementary Material. All microbial isolates used were stored long term at –80 °C on Microbank™ beads and revived prior to use on appropriate solid agar. *C. albicans* SC5314 was sub-cultured on Sabouraud dextrose agar, whereas *S. aureus* Newman, *S. aureus* SH1000 and *Pseudomonas aeruginosa* PA14 were all streaked onto Luria–Bertani (LB) agar. Fungal plates were incubated aerobically at 30 °C for 24–48 h, and bacterial plates were incubated aerobically at 37 °C for 24 h.

Next, overnight cultures in liquid media were prepared by inoculating a single colony into either 10 ml of yeast peptone dextrose medium (1% yeast extract, 2% peptone, 2% dextrose) for *C. albicans*, or LB broth for the bacterial strains. Cultures were incubated with shaking at 200 r.p.m. at 30 °C for *C. albicans* or at 37 °C for all bacteria. Following overnight culture, cells were harvested by centrifugation at 3,500 r.p.m. for 5 min, washed twice in PBS (10 mM phosphate buffer, 2.7 mM KCl, 137 mM NaCl, pH 7.4), and resuspended in 10 ml of PBS.

Fungal cell concentrations were determined by haemocytometer counts using a 1 : 100 dilution (10 µl resuspended, washed culture in 990 µl PBS), with final normalized counts expressed as c.f.u. ml^−1^ for the starting suspension. Bacterial suspensions were standardized to 1×10⁸ c.f.u. ml^−1^ by adjusting the OD at 600 nm to an absorbance of 0.6, with cell counts previously determined using Miles and Misra technique [31]. Standardized cultures were subsequently used to generate either planktonic or biofilm populations in appropriate media.

### Kinetic growth curve

The inhibitory effects of KHS101 hydrochloride (herein known as KHS101), darapladib and polygodial on planktonic cells were evaluated against *S. aureus* Newman and SH1000 strains. All three compounds were chosen based on prior screening of the Tocriscreen™ bioactive compound library, where they demonstrated varying degrees of antifungal activity [29]. To determine whether this activity extended beyond fungi, they were subsequently evaluated for antibacterial effects against *S. aureus* using kinetic growth curves. Each compound was dissolved in 100% DMSO (wt/v) to prepare 100 mM stock solutions, which were aliquoted into sterile microcentrifuge tubes and stored at −20 °C until use.

Cell suspensions were prepared following standardization protocols as above, with bacteria adjusted to 2×10⁵ cells ml^−1^ in LB broth. Stocks were prepared at double the required concentration (128 µg ml^−1^) in LB broth and serially diluted twofold across 96-well round-bottom microtitre plates. For each dilution series, 200 µl of the antimicrobial solution was added to the first column, followed by serial doubling dilutions in LB broth. Standardized microbial suspensions (100 µl) were then added to each well, yielding a final volume of 200 µl well^−1^.

Plates were incubated aerobically at 37 °C for 24 h with shaking at 200 r.p.m. in an orbital shaker. Growth was monitored using a Cerrillo™ Stratus microplate reader with OD absorbance measurements at 600 nm recorded every 2 h. Growth curves were generated by plotting mean OD_600_ values over time. Positive (cells only) and negative (media only) controls were included in all assays. Endpoint OD_600_ values at 24 h were normalized to the positive control, and compounds at concentrations achieving ≥50% or ≥90% reduction in growth were considered to display inhibitory activity. All kinetic growth curves were conducted with two biological replicates run from independent cultures, averaged from four technical replicates.

### Planktonic MIC testing

*S. aureus* planktonic MICs (pMICs) against flucloxacillin and gentamicin were tested using the European Committee for Antimicrobial Susceptibility Testing (EUCAST) standard pMIC protocol [32]. Briefly, overnight cultures of Newman and SH1000 *S. aureus* were standardized to 1×10^8^ c.f.u. ml^−1^. These were then further diluted to 2×10^5^ c.f.u. ml^−1^ in LB broth, and 100 µl well^−1^ of the suspension was added to a round-bottom polystyrene 96-well plate. A serial dilution series of each treatment, flucloxacillin and gentamicin, was then added to each well (100 µl well^−1^). Plates were incubated at 37 °C under static conditions for 24 h, and pMICs were visually determined following incubation.

### Biofilm formation

Biofilms were created either in a serum-containing hydrogel-cellulose matrix three-dimensional system for KHS101-CAP therapy, on a 13 mm Nunc™ Thermanox™ coverslip for scanning electron microscopy (SEM) or on the bottom of 96-well flat-bottomed plates for the sensitization assays. The hydrogel-cellulose matrix system was used as a wound-relevant biofilm model initially, as previously described [33], to support growth of mono-species and polymicrobial communities.

Hydrogels were prepared following a previously published method [33, 34]. In short, the formulation contained 10% 3-sulfopropyl acrylate potassium salt, 0.95% v/v poly(ethylene glycol) diacrylate, 0.01% v/v 1-hydroxycyclohexyl phenyl ketone and 50% heat-inactivated horse serum, with 2×PBS added to achieve the final volume. A total of 2 ml of hydrogel mixture was dispensed per well in 12-well flat-bottomed plates and polymerized for 1 h under 366 nm UV light in a class II laminar flow hood. Polymerized hydrogels were stored at 4 °C and used within 1 week.

Mono-species and triadic biofilms were established on sterile cellulose matrices (1.25×1.25 cm²) placed within the hydrogel system. Standardized microbial suspensions of final concentrations of 1×10^6^ c.f.u. ml^−1^ were inoculated onto cellulose matrix substrata and incubated at 37 °C with gentle agitation at 180 r.p.m. for 2 h to allow cell attachment. The matrices were then washed once with PBS to remove non-adherent cells and transferred onto the hydrogel surface for 24 h aerobic incubation at 37 °C to permit biofilm formation. Negative cellulose matrix controls without inoculum were included in all experiments to assess sterility.

For biofilm formation on coverslips and 96-well plates, all microbial organisms were standardized to a final working concentration of 1×10^6^ c.f.u. ml^−1^. Biofilms on coverslips were formed following careful placement of substrata into 24-well plates, prior to addition of 500 µl of standardized suspension. In 96-well plates, 200 µl of the standardized microbial suspensions were dispensed into each well using a multi-channel pipette. The inoculated plates were then incubated aerobically under static conditions at 37 °C for a period of either 4 or 24 h to allow for biofilm formation.

### KHS101-CAP treatment regimens

For biofilm studies, a working concentration of KHS101 of 32 µg ml^−1^ was selected based on kinetic growth curve results. Following hydrogel-biofilm formation after 24 h as above, matrices were washed with PBS to remove non-adherent cells before treatment. Biofilms were exposed to 32 µg ml^−1^ of KHS101 for 5 min, either alone or in combination with CAP. CAP monotherapies were also conducted as previously published [35]. In brief, this involved a custom-built multi-jet argon plasma device comprising six plasma jets arranged in a conical/rectangular configuration and was operated using argon gas. CAP treatments were performed under standard ambient laboratory conditions. Ambient temperature and relative humidity were not recorded; however, all experiments were conducted using a fixed total flow rate of 6 standard l min^−1^, with an applied voltage of 8 kV and a frequency of 23.5 kHz. The same CAP operating conditions were used in our previous study, in which plasma-generated H_2_O_2_ was quantified and identified as a major long-lived reactive species generated by this device [35]. Under treatment conditions, H_2_O_2_ concentrations were ~1 mM after 5 min exposure. These values also informed the 1 mM H_2_O_2_ concentration used in the mechanistic comparison experiments. Full physical characterization of the plasma device, including electrical and optical diagnostics, has been reported previously [36].

After treatment with KHS101, all biofilms, including the untreated (UT) controls, were neutralized with 500 µl of Dey–Engley neutralizing broth, then incubated for 15 min at 37 °C aerobically to deactivate any treatment effect. The same neutralizer was added to all UT controls for consistency. Following deactivation, the neutralizing broth was removed by washing in 500 µl of PBS, and for dual therapies or CAP monotherapy, CAP exposure was undertaken with matrices positioned 1 cm from the jet outlets. The cellular substrate was inverted halfway through each treatment to ensure sufficient CAP coverage of both sides of the matrix. All biofilms were then placed into a bijoux containing 1 ml of sterile PBS prior to assessment of biofilm viability.

### Assessment of biofilm viability

Biofilm biomass was detached from cellulose matrices by sonication in 1 ml of PBS at 35 kHz for 10 min, followed by 30 s of vortexing. The resulting suspension was divided into two aliquots: one treated with 2.5 µl of 10 mM propidium monoazide (PMA) to selectively penetrate dead or membrane-compromised cells, and one minus PMA as a control. PMA treatment was used as previously described for this hydrogel-cellulose matrix system without modification [33]. Samples were incubated in the dark for 10 min, then photoactivated on ice under halogen light (650 W, 5 min at a distance of 20 cm). All samples were stored at −20 °C until DNA extraction.

DNA was extracted using a bead-beating protocol combined with the QIAamp DNA Mini Kit, with modifications to enhance microbial lysis as previously described [33, 37]. Briefly, cells were pelleted and resuspended in ATL buffer with proteinase K, incubated at 56 °C for 20 min, then lysed further with AL buffer at 70 °C for another 10 min. Samples underwent bead-beating (3×30 s) using a Fisherbrand™ Bead Mill 24 Homogenizer. Following ethanol precipitation, nucleic acids were purified using spin columns with wash buffers and eluted in 100 µl AE buffer. DNA from pure *S. aureus* cultures, serially diluted from 10^8^ to 10^3^ c.f.u. ml^−1^, were extracted in parallel to generate standard curves.

Quantification of live and dead populations was performed by SYBR Green-based qPCR on a StepOnePlus™ system using the following primer sets: forward primers 5′−3′ ATTTGGTCCCAGTGGTGTGGGTAT and reverse primers 5′–3′ GCTGTGACAATTGCCGTTTGTCGT [33]. Amplification conditions were as follows: 50 °C (2 min), 95 °C (2 min), then 40 cycles of 95 °C (10 s) and 60 °C (30 s). Colony-forming equivalents per ml (CFE ml^−1^) were interpolated from standard curves. All experiments were performed in triplicate across three independent runs (*n*=9). All three biological replicates represent independent experiments performed on separate occasions from independently prepared cultures.

### Combination analysis of KHS101 and CAP

Combination effects between KHS101 and CAP were assessed using Bliss independence and highest single agent (HSA) analyses. To account for variation in total biofilm recovery between treatments, analyses were performed using proportional viability values, calculated as viable cells divided by total cells and normalized to the corresponding UT control for each biological replicate. Bliss analysis was used to determine whether the observed dual-treatment effect exceeded the effect predicted from the two monotherapies, while HSA analysis assessed whether the dual treatment outperformed the most effective monotherapy.

### Transmission electron microscopy for assessment of ultrastructural changes

Overnight suspensions of *S. aureus* cells were pelleted and washed with PBS, then treated with 32 µg ml^−1^ KHS101 for 5 min. Cells were pelleted and gently washed with PBS and fixed overnight at 4 °C in a solution containing 4% paraformaldehyde, 2.5% glutaraldehyde and 0.15 M sodium cacodylate buffer supplemented with 0.15% Alcian blue. Samples were subsequently processed at the University of Glasgow Imaging Facility. In brief, following three washes in 0.15 M sodium cacodylate buffer containing 2% sucrose, cells were post-fixed in 1% osmium tetroxide for 1 h, washed and contrast-stained with 0.5% aqueous uranyl acetate for 1 h in the dark. Samples were dehydrated through a graded ethanol series, transitioned through propylene oxide and infiltrated with Araldite/Epon 812 resin before polymerization at 60 °C for 48 h. Ultrathin sections (50–60 nm) were prepared using a Leica Ultracut UCT ultramicrotome, collected on formvar-coated copper grids and post-stained with uranyl acetate and Reynolds lead citrate. Transmission electron microscopy was performed using a JEOL1200EX microscope operated at 80 kV, with images acquired at ×15,000 and ×40,000 magnifications.

### Scanning electron microscopic profiling of biofilms

To visualize *S. aureus* Newman and SH1000 biofilms on coverslips, SEM was employed. Biofilm-containing coverslips±KHS101 treatment were washed twice with PBS and fixed for 24 h in a solution containing 2% glutaraldehyde, 2% paraformaldehyde, 0.15% (w/v) Alcian blue and 0.15 M sodium cacodylate buffer (pH 7.4). Following fixation, biofilms were rinsed three times in sodium cacodylate buffer, then incubated for 1 h in a 1 : 1 mixture of osmium tetroxide and sodium cacodylate buffer. Samples were then washed in distilled water for three successive 10 min steps and contrast-stained with 0.5% uranyl acetate. Dehydration was carried out using a graded ethanol series (30–90%), followed by four 10 min incubations in absolute ethanol. Dried samples were prepared using hexamethyldisilizane under desiccation for 24 h, mounted on stainless steel stubs, and coated with a gold/palladium layer by sputter coating. Imaging was conducted using a JEOL JSM-IT100 InTouch™ scanning electron microscope.

### Fluorescence microscopy for assessment of membrane integrity

*S. aureus* Newman and SH1000 grown overnight in LB broth were pelleted and washed with PBS, then standardized to 1×10^8^ c.f.u. ml^−1^. Following this, cells were treated with 32 µg ml^−1^ KHS101 for 5 min, with UT controls used as comparators. Cells were pelleted and washed with PBS to remove residual treatment, then were stained with SYTO-9 and propidium iodide (PI) in 1 ml PBS for 15 min, protected from light. Following staining, cells were pelleted again, washed to remove dye then fixed with 2% formalin for 1 h. Cells were centrifuged and washed for one final step, before resuspension in 500 µl. Cells were then imaged by dispensing 10 µl on a glass slide, with a coverslip placed on top, then visualized using the EVOS M5000 imaging system. Images were captured at ×10 magnification.

### Assessment of biofilm metabolic activity

To assess biofilm metabolic activity for the sensitization assays, overnight cultures of *S. aureus* Newman and SH1000 biofilms were formed for 4 h as described above. It is worth noting that 4 h biofilms were selected for metabolic assays as an early, metabolically active biofilm model suitable for AlamarBlue^®^-based screening. These experiments were intended as mechanistic sensitization assays to explore the dual actions of compounds with H_2_O_2_, rather than direct comparators to the 24 h biofilm model used for KHS101 +CAP efficacy testing as described above.

Following growth, the 4 h biofilms were then treated with KHS101 (starting conc. 128 µg ml^−1^), flucloxacillin (starting conc. 8,000 µg ml^−1^) or gentamicin (starting conc. 128 µg ml^−1^) that were serially double-diluted, in combination with H_2_O_2_ at a constant concentration of 1 mM; equivalent concentrations of which were produced by 5 min CAP therapy [35]. Biofilms were treated with either H_2_O_2_ alone, KHS101 or antibiotic alone or a combination of KHS101 or antibiotic and H_2_O_2_ together for 24 h. In addition, biofilms were pre-treated with H_2_O_2_ for 10 min followed by antibiotic for 24 h or treated with antibiotics for 24 h followed by 10 min post-treatment with H_2_O_2_. Following treatment, a neutralization step was performed by adding 200 µl of 5% sodium thiosulphate for 15 min incubation at room temperature to deactivate the treatment effect. Sodium thiosulphate was also applied to negative and positive controls to normalize results. Sodium thiosulphate was used over Dey–Engley broth for these experiments as the neutralizer was more compatible with AlamarBlue^®^-based metabolic readouts. Sodium thiosulphate was also applied to positive and negative controls to account for any potential effect on the AlamarBlue^®^ readout.

Following this, the neutralizer was removed, and biofilms were washed once with 200 µl PBS. The effects of each treatment were then assessed using the AlamarBlue^®^ metabolic assay as previously described [37]. In brief, the reagent was diluted 1 : 10 with LB medium, and 100 µl of this solution was added to each well under dark conditions. The plates were then incubated at 37 °C aerobically for 20 min. Following colour change in the UT controls (blue to pink), 75 µl from each well was transferred to a new 96-well flat-bottom microtiter plate for fluorescence measurement. Fluorescence values were read using a BMG Labtech FLUOstar plate reader at 570 nm (excitation) and 600 nm (emission). Experiments were performed in triplicate, with three technical repeats per experiment as above (*n*=9).

### Plate count assay

To quantify additive effects of H_2_O_2_ on cellular viability, plate count assay tests were performed for a selected concentration of each compound (KHS101, flucloxacillin, gentamicin) and each H_2_O_2_ combinational therapy. The compound concentrations displaying metabolic activity above 50% when administered alone were selected as follows; KHS101 at 8 µg ml^−1^, flucloxacillin at 250 µg ml^−1^ and gentamicin at 128 µg ml^−1^. Following different treatment regimens and neutralization as above, biofilms were scraped using a sterile wooden applicator stick and drop plated onto Tryptic Soy Agar plates and viable plate counting was performed as previously described [38]. Log_10_ c.f.u. ml^−1^ values were calculated for each sample following overnight incubation. Experiments were performed in triplicate, with three technical repeats per experiment as above (*n*=9).

### Ethics and biosafety

All bacterial and fungal work was conducted under institutional Containment Level 2/Biosafety Level 2 conditions in accordance with University of Glasgow biological safety procedures and approved local risk assessments within the College of Medicine, Veterinary and Life Sciences. No human or animal subjects were used throughout the duration of this study, therefore no ethical approval from an ethics committee was required for experimental work.

### Statistical analysis

All statistical analyses, graphs, and distribution assessments were conducted using GraphPad Prism version 11.0.2 (GraphPad, San Diego, CA, USA). Data normality was first evaluated using the D'Agostino–Pearson omnibus test for CAP-KHS101 biofilm studies, and Shapiro–Wilk test for plate count assays. All data were non-normally distributed, therefore, Kruskal–Wallis tests followed by Dunn’s post-tests were employed. Differences were considered statistically significant if *P*<0.05.

## Results

**Fig. 1.**
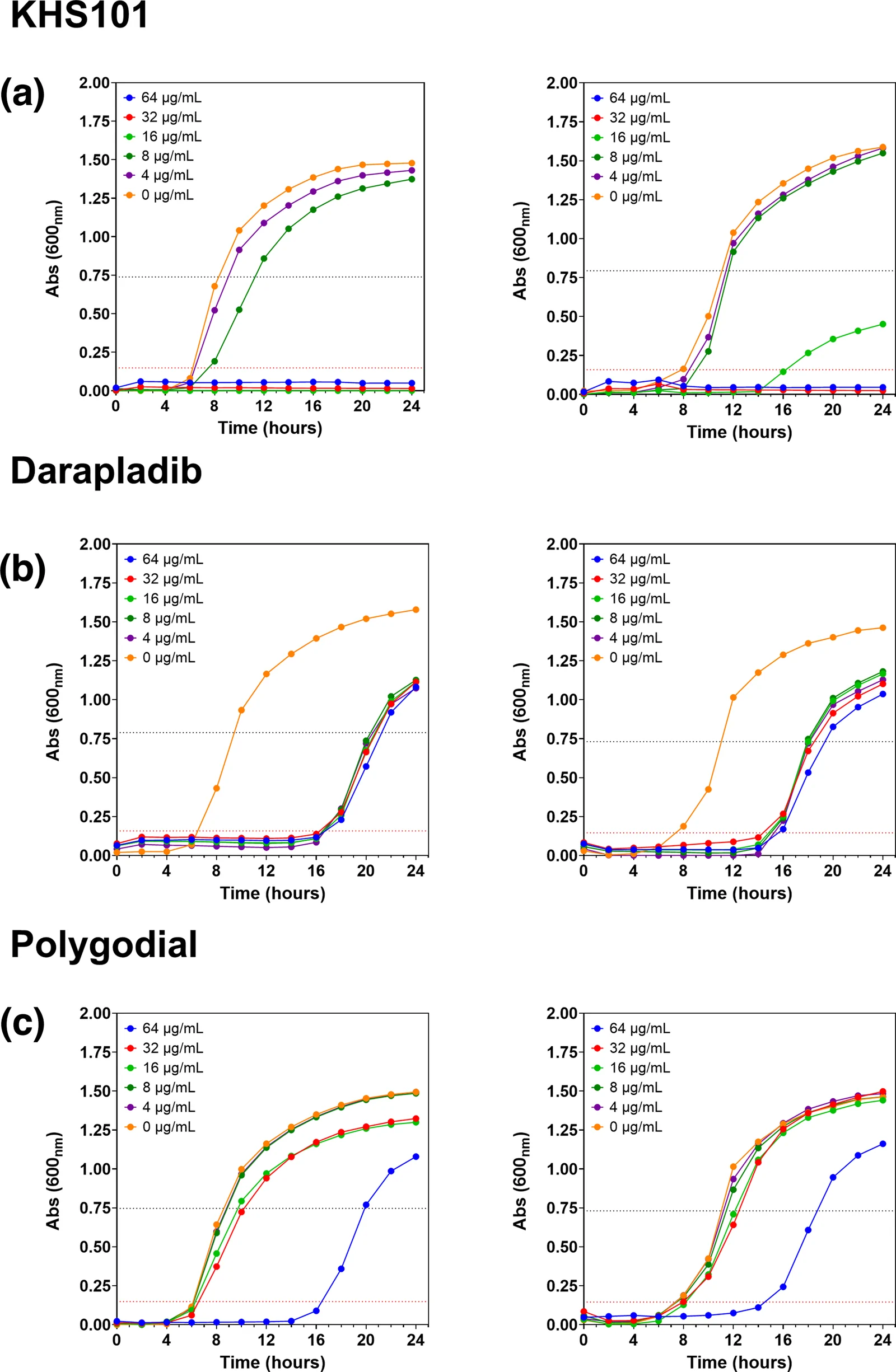
Twenty-four hour growth curve of *S. aureus* Newman and SH1000 strains treated with KHS101 hydrochloride, darapladib and polygodial. Planktonic cells of *S. aureus* Newman (left panels) and SH1000 (right panels) were grown in LB broth in a Cerillo microplate reader device with shaking at 200 r.p.m., at 37 °C. Suspensions were treated with 0–64 µg ml^−1^ concentrations of KHS101 hydrochloride (a), darapladib (b) and polygodial (c) for 24 h, read at 1 h intervals. The curves show the means of OD at 600 nm from four technical replicates across two biological replicates (*n*=8). 0 µg ml^−1^ refers to the UT control. Dotted lines denote 50% reduction (black) and 90% reduction (red) in absorbance at 24 h. Data plotted as mean values only; error bars were omitted for visual clarity of data presentation.

To start, the inhibitory effects of KHS101, darapladib and polygodial were evaluated against *S. aureus* Newman and SH1000 strains grown in planktonic suspension over 24 h. All three compounds have previously been shown to possess killing activity against different fungal biofilms [29], but their antibacterial effects are unknown. Both *S. aureus* strains demonstrated sensitivity to the KHS101 compound at concentrations of 32 µg ml^−1^ and 64 µg ml^−1^ after 24 h, respectively (Fig. 1a). However, KHS101 was more effective against *S. aureus* Newman than SH1000, with some reduction in growth at 16 µg ml^−1^. The UT control had a mean OD of 1.47 after 24 h, with 16 µg ml^−1^ with no growth seen across the time course of the experiment. *S. aureus* SH1000 showed some susceptibility to the compound at 16 µg ml^−1^, with no growth until ~14 h, but only 71.6% inhibition observed after 24 h (mean OD of 1.58 for UT vs mean OD of 0.45 at 16 µg ml^−1^). Both 32 µg ml^−1^ and 64 µg ml^−1^ concentrations displayed complete inhibition (>99.0%).

*S. aureus* Newman and SH1000 displayed moderate sensitivity to darapladib at all concentrations tested across 16 h, before the micro-organisms largely recovered from any antibacterial effect to the compound after 24 h (Fig. 1b). Polygodial had similar minimal endpoint effects on the two *S. aureus* strains. At concentrations ranging from 4 to 32 µg ml^−1^, growth kinetics were comparable with UT controls. At the highest concentration, 64 µg ml^−1^ inhibited growth to ~14–16 h for both micro-organisms, before recovery by 24 h (Fig. 1c). These results highlight that KHS101 has the most promising antibacterial effects against *S. aureus* and as a result was selected for further testing as a candidate for dual therapy to break bacterial tolerance to CAP [35].

**Fig. 2.**
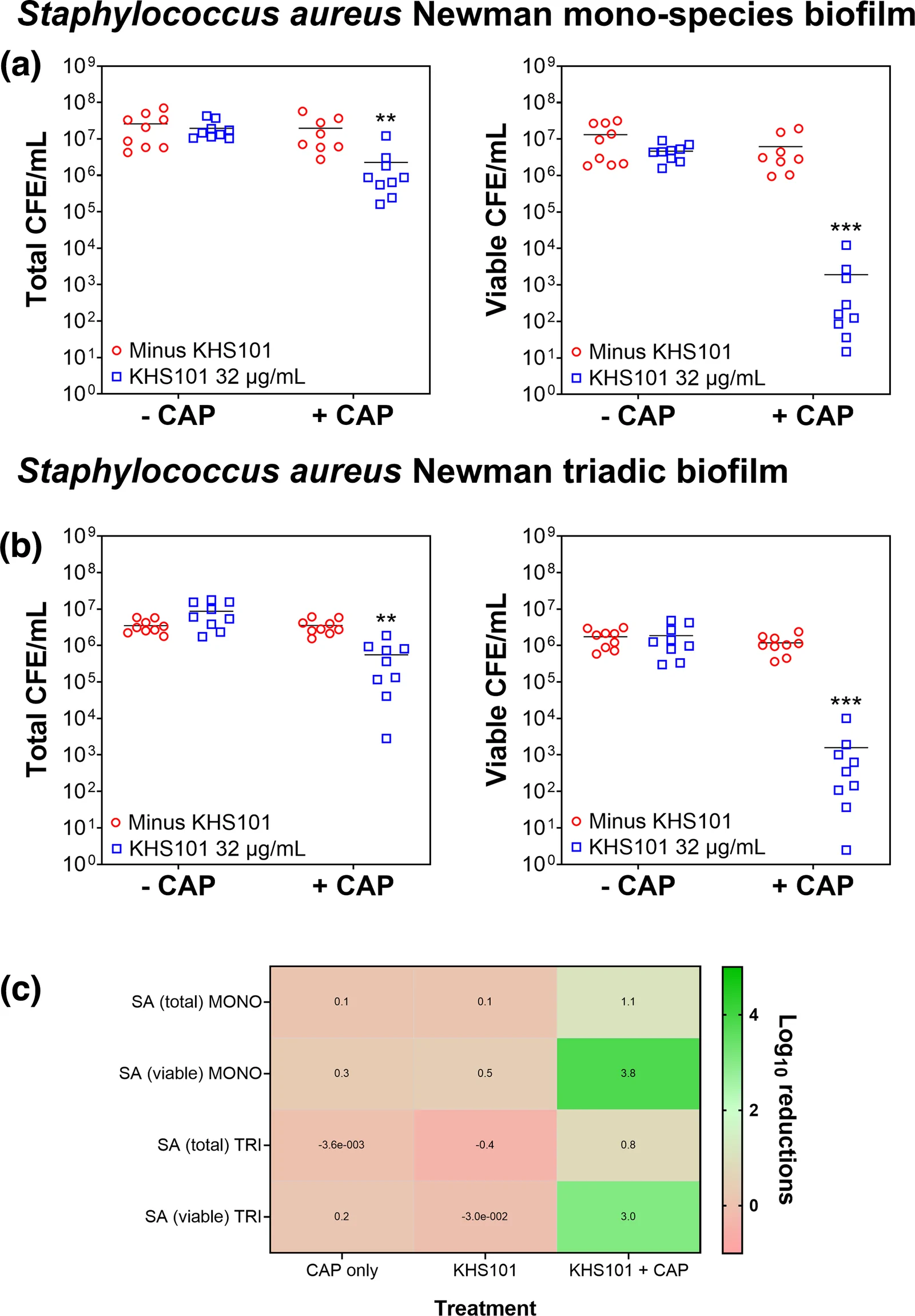
Molecular analysis of *S. aureus* Newman biofilm models following treatment with KHS101 alone or with CAP dual therapy. *S. aureus* Newman mono-species biofilms were grown for 24 h within the hydrogel-cellulose matrix system and treated with KHS101 (32 µg ml^−1^: blue dots) for 5 min alone or with an additional 5 min therapy by CAP after the neutralization (Dey–Engley broth for 15 min at 37 °C). Controls minus KHS101±CAP were used for comparison (red dots). Neutralizer had no impact on cellular viability. Total and viable CFE ml^−1^ counts were quantified using live/dead qPCR (a). Triadic polymicrobial biofilms composed of *S. aureus* Newman, *C. albicans* SC5314 and *P. aeruginosa* PA14 were established under the same conditions and subjected to identical treatments. Total and viable cell counts for *S. aureus* only are shown (**b**). Heatmap representation of log_10_ reductions in total and viable *S. aureus* counts compared to UT controls when grown as mono-species or triadic biofilms. Results representative of mean values from *n*=9 (three technical replicates from three biological experimental repeats). Non-normally distributed data were analysed by Kruskal–Wallis with Dunn’s tests to determine the *P*-value for multiple comparisons. * Indicates statistically significant differences compared to the minus treatment controls (**P<0.01, ***P<0.001).

**Fig. 3.**
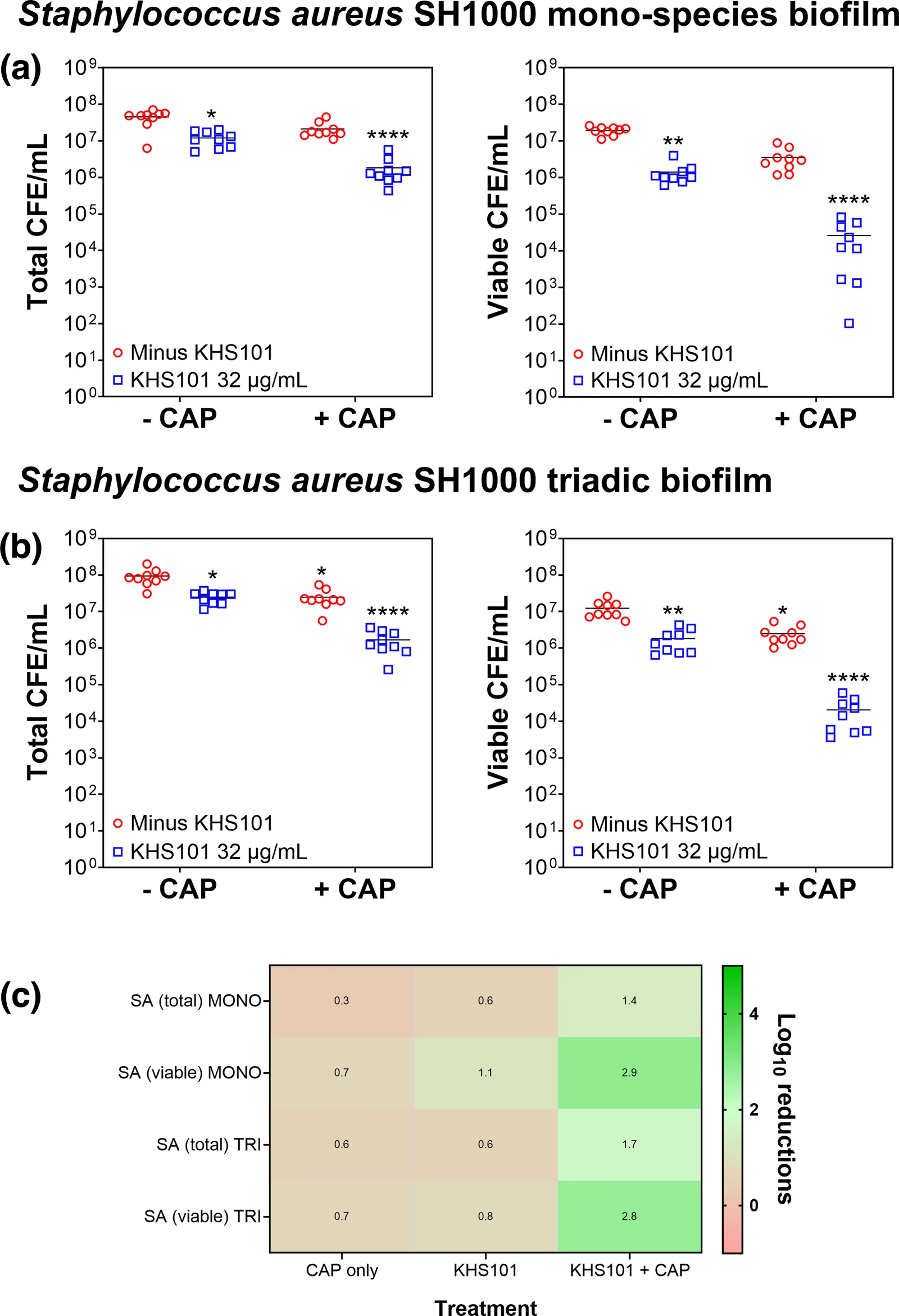
Molecular analysis of *S. aureus* SH1000 biofilm models following treatment with KHS101 alone or with CAP dual therapy. Twenty-four hour mature *S. aureus* SH1000 biofilms grown in the hydrogel-cellulose matrix system were treated with KHS101 (32 µg ml^−1^: blue dots) ± CAP, each for 5 min. Between treatments, KHS101 activity was neutralized with Dey–Engley broth for 15 min. Experiments were conducted against *S. aureus* SH1000 mono-species biofilms (a) or when grown together with *C. albicans* SC5314 and *P. aeruginosa* PA14 in a triadic model (b). Data presented as CFE ml^−1^ quantified for each biofilm following treatment using live/dead qPCR. Results are representative of mean values from *n*=9 (three technical replicates from three biological experimental repeats). Panel (**c**) shows log_10_ reductions of total and viable cell counts in a heatmap compared to the UT controls (minus KHS101 and CAP). Non-normally distributed data were analysed by Kruskal–Wallis with Dunn’s tests to determine the *P*-value for multiple comparisons. * Indicates statistically significant differences compared to the minus treatment controls (*P<0.05, **P<0.01, ***P<0.001, ****P<0.0001).

We have previously shown that mono-CAP treatment was ineffective in killing both of these *S. aureus* strains grown as mono-species or within multi-species biofilm models [35]. Given the potent effects of 32 µg ml^−1^ KHS101 against planktonic cells, we proposed that short-term treatment with KHS101 (~5 min), followed by CAP therapy, may break this previously reported tolerance to the agents produced via CAP. Treatments were conducted as monotherapies for 5 min or in combination with CAP for an additional 5 min. Results displayed in Figs 2 and 3 show the total and viable cell counts of *S. aureus* Newman and SH1000 following mono- and dual therapies with the above treatments.

In *S. aureus* Newman mono-species biofilms (Fig. 2a, c), dual therapy of KHS101 with CAP was an effective treatment intervention. KHS101 monotherapy produced only a modest 0.1-log_10_ reduction in total cell counts (from 2.59×10^7^ to 1.96×10^7^ CFE ml^−1^) and a 0.5-log_10_ decrease in viable cells (to 4.66×10^6^ c.f.u. ml^−1^) when compared to UT controls. When in combination with CAP, efficacy was markedly enhanced, achieving a 1.0-log_10_ reduction in total cells (**P=0.003; to 2.56×10^6^ c.f.u. ml^−1^) and a striking 3.8-log_10_ reduction in viable cells (***P=0.0001; from 1.31×10^7^ to 1.90×10^3^ c.f.u. ml^−1^). This was further evidenced when accounting for proportional viability, with KHS101 alone reducing proportional viability from 50.7% in UT, 31.4% in CAP only and 23.7% in KHS101 only to 0.08% in dual therapy. Similar trends were observed in triadic biofilms containing *S. aureus* Newman (Fig. 2b, c), where KHS101 plus CAP lowered total cells by 0.8-log_10_ (**P=0.0061; to 5.56×10^5^ CFE ml^−1^) and viable cells by 3.0-log_10_ (***P=0.0002; to 1.58×10^3^ CFE ml^−1^). Reductions in proportional viability also strengthened these observations. KHS101 with CAP decreased biofilm viability from 49.6% in UT, 33.4% in CAP only and 21.56% in KHS101 only to 0.28% in dual therapy.

Overall, the same anti-biofilm dual therapy effect was observed for the SH1000 strain (Fig. 3). Conversely to the Newman strain, KHS101 treatment alone reduced total counts from 4.54×10^7^ to 1.21×10^7^ CFE ml^−1^ (*P=0.0233; 0.6-log_10_) and viable counts from 1.95×10^7^ to 1.41×10^6^ CFE ml^−1^ (**P=0.0022; 1.1-log_10_). Similarly to the other strain, the antimicrobial effect was more pronounced in dual therapies, with total and viable counts of 1.84×10^6^ CFE ml^−1^ (****P<0.0001; 1.4-log_10_ reduction) and 2.62×10^4^ (****P<0.0001; 2.9-log_10_ reduction), respectively. The antimicrobial activity of the therapies was further evidenced in the proportional viability post-treatment. UT controls were 43.9% viable, KHS101-treated biofilms were 11.7%, CAP-treated biofilms 16.8%, whilst dual therapy reduced viability to 1.42%. Comparable results were observed for *S. aureus* SH1000 grown as part of a triadic consortia. Reductions of 1.7-log_10_ (****P<0.0001) and 2.8-log_10_ (****P<0.0001) were observed in total and viable counts in three-species biofilms treated with KHS101 and CAP, respectively. These experimental results from the triadic models for both strains highlight that other microbial species within the biofilm conferred no protective effect on *S. aureus* against KHS101 and CAP dual therapy. Taken together, these results indicate that KHS101 when used prior to CAP may offer a suitable candidate compound to break the *S. aureus* tolerance to CAP alone.

From the results in Figs 2 and 3, KHS101 combined with CAP produced enhanced antimicrobial activity compared with either monotherapy. To formally assess this interaction, combination effects were analysed using Bliss independence and HSA models based on proportional biofilm viability. Bliss analysis indicated that KHS101 +CAP exceeded the predicted additive effect in all conditions, with mean ΔBliss values ranging from 0.140 to 0.351. HSA analysis further demonstrated that the dual treatment outperformed the most active monotherapy across all biofilm models, with mean ΔHSA values ranging from 0.263 to 0.472. Together, these analyses support enhanced combined antimicrobial activity between KHS101 pre-treatment and CAP exposure, consistent with the possibility that KHS101 may sensitize biofilm cells and/or its structure to subsequent CAP therapy.

**Fig. 4.**
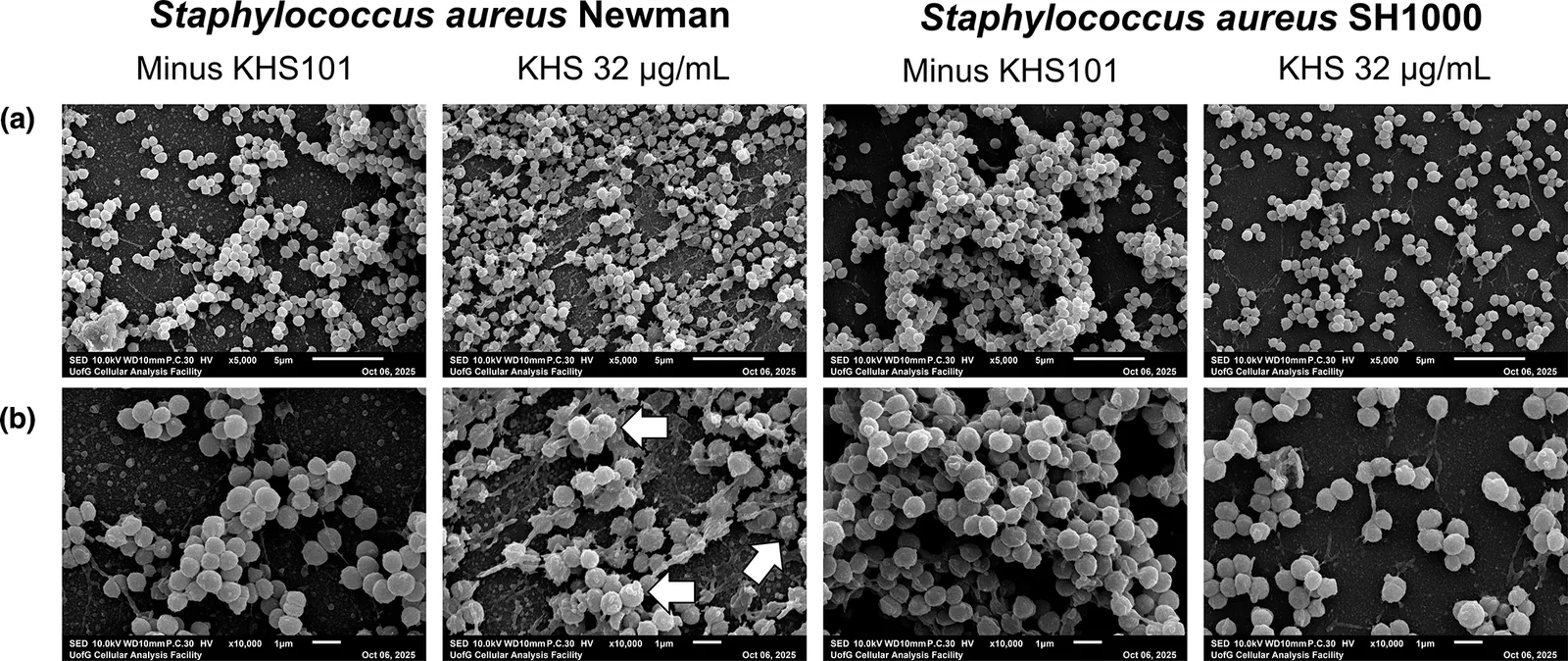
SEM imaging of *S. aureus* biofilms pre- and post-treatment with KHS101. Biofilms were treated for 5 min with KHS101 then visualized using a JEOL JSM-IT100 InTouch™ scanning electron microscope. UT controls (minus KHS101) were imaged for comparative purposes. Images were taken at ×5,000 (a) and ×10,000 (b) magnifications. Changes to the cellular morphologies of *S. aureus* Newman post-treatment with KHS101 are highlighted by white arrows, evidenced by membrane blebbing. These blebs appeared as small (<0.25 µm) protrusions from the cellular surface.

Next, to assess the impact of KHS101 on the biofilm at a morphological level, SEM images were taken of UT biofilms and those treated with KHS101 for 5 min. Images revealed that UT biofilms displayed the expected coccoid morphology with smooth, intact cell surfaces and dense cellular clustering at both ×5,000 and ×10,000 magnifications. Conversely, surface irregularities and membrane blebbing were evident in the treated biofilms, particularly for the *S. aureus* Newman strain but absent from the SH1000 strain (Fig. 4). To further investigate these potential KHS101-mediated surface irregularities within the biofilms, but this time at a subcellular level, transmission electron microscopy (TEM) imaging was used (Fig. S1). TEM analysis indicated that short-term exposure to KHS101 did not result in cellular death and was associated with little to no ultrastructural changes. KHS101-treated cells retained intact cell envelopes, preserved septal architecture and no evidence of cytoplasmic leakage or membrane rupture. Finally, to further explore the effects of KHS101 on the *S. aureus* cells, fluorescence microscopy was conducted using SYTO-9 and PI stains (Figs S2 and S3). KHS101-treated *S. aureus* Newman and SH1000 cells showed a subtle increase in PI-associated red fluorescence compared with UT controls, indicating some changes to membrane permeability following exposure to the compound, observations not picked up by TEM. Collectively, these results indicate that the impact of KHS101 on *S. aureus* is limited under these conditions, with no evidence of substantial antimicrobial activity when administered as a monotherapy. Instead, its effects appear to be non-lethal and associated with subtle stress-related changes that may increase susceptibility to subsequent CAP treatment.

**Fig. 5.**
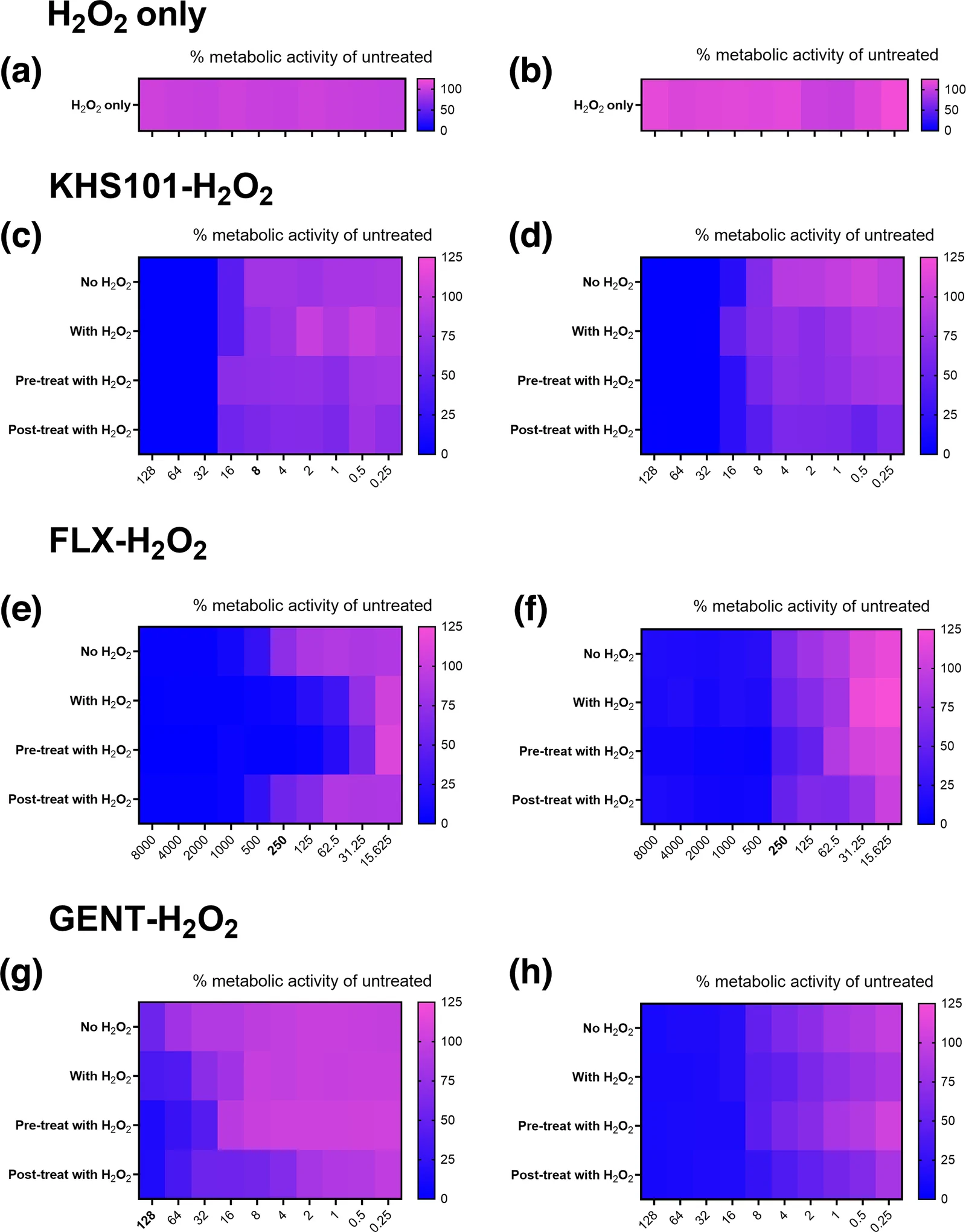
Investigations into the mechanism of action of KHS101 in combination with H_2_O_2_. H_2_O_2_ treatment at 1 mM alone had no effect against both 4 h *S. aureus* biofilms (a and b). Newman biofilms (c) and SH1000 biofilms (d) were also treated with the following; KHS101 only at different concentrations (0.25–128 µg ml^−1^), KHS101 with 1 mM H_2_O_2_, KHS101 prior to treatment with 1 mM H_2_O_2_ (pre-treat) and KHS101 after treatment with 1 mM H_2_O_2_ (post-treat). For comparative purposes with KHS101, flucloxacillin (e and f: FLX) and gentamicin (g and h: GENT) were selected as candidate antibiotics and tested in the same way alongside H_2_O_2_. Following treatments and neutralization, AlamarBlue^®^ assay was utilized to assess metabolic activity of the biofilms. Data presented as percent metabolic activity relative to the UT controls, with values of 100% (bright pink colour) equating to no antimicrobial effect, and 0% (dark purple colour) corresponding to no biofilm metabolic activity.

Next, in Fig. 5, experiments were conducted to investigate whether H_2_O_2_, a major long-lived reactive species produced by our CAP device, could reproduce or contribute to the enhanced antimicrobial activity observed with KHS101 +CAP. A concentration of 1 mM H_2_O_2_ was selected as a major component produced by our CAP device, as previously reported [35]. To determine whether the observed H_2_O_2_-mediated effects were specific to KHS101 or reflected a more general oxidative stress response by the micro-organisms, two conventional antibiotics were included as comparators. Flucloxacillin and gentamicin were selected due to their distinct and well-characterized mechanisms of action targeting cell wall synthesis and protein synthesis, respectively. Initially, these antibiotics were first tested for their pMICs against both strains, which ranged from 0.125 to 0.50 µg ml^−1^ and 2–4 µg ml^−1^ for flucloxacillin and gentamicin, respectively (Table S2). For the biofilms, cells were exposed to KHS101 or antibiotics alone or in combinations delivered simultaneously, as a pre- or post-treatment. Metabolic activity was expressed relative to UT controls. It is noteworthy that H_2_O_2_ at 1 mM alone had no effect on the biofilm metabolic activity compared to the controls (Fig. 5a, b).

Importantly, H_2_O_2_ at a CAP-equivalent concentration did not enhance the antimicrobial effect of KHS101 against either *S. aureus* strain, but did potentiate antibiotic activity in a strain-dependent manner. KHS101 concentrations at 32 µg ml^−1^±1 mM H_2_O_2_ inhibited metabolic activity at >90% for both strains (Fig. 5c, d). This suggests that reactive species generated by CAP other than H_2_O_2_, such as short-lived RONS, may play a more prominent or combinational role in mediating the enhanced activity of KHS101 observed during CAP treatment as documented in Figs 2 and 3. Conversely, both antibiotics showed a decrease in metabolic activity at lower concentrations of each antibiotic when in combination with 1 mM H_2_O_2_ compared to the drug alone for *S. aureus* Newman (Fig. 5e, g). In the case of flucloxacillin, at 250 µg ml^−1^ when antibiotic alone was administered metabolic activity was ~70%. In contrast, when in combination with 1 mM H_2_O_2_ a noticeable decrease in metabolic activity to 8% for simultaneous treatment, 2% for pre-treatment and 55% for post-treatment, were observed respectively. Similar effects can be observed for gentamicin as well, where antibiotic alone shows a metabolic activity of ~55%, with a decrease to 37% for simultaneous treatment, 15% for pre-treatment, and 16% for post-treatment. Moreover, the effect appears to be potentiated when pre-treatment with 1 mM H_2_O_2_ was performed. Interestingly, little to no enhanced effects were observed for *S. aureus* SH1000 for both antibiotics (Fig. 5f, h), suggesting that there might be strain-specific features influencing the success of these combinatorial treatments.

**Fig. 6.**
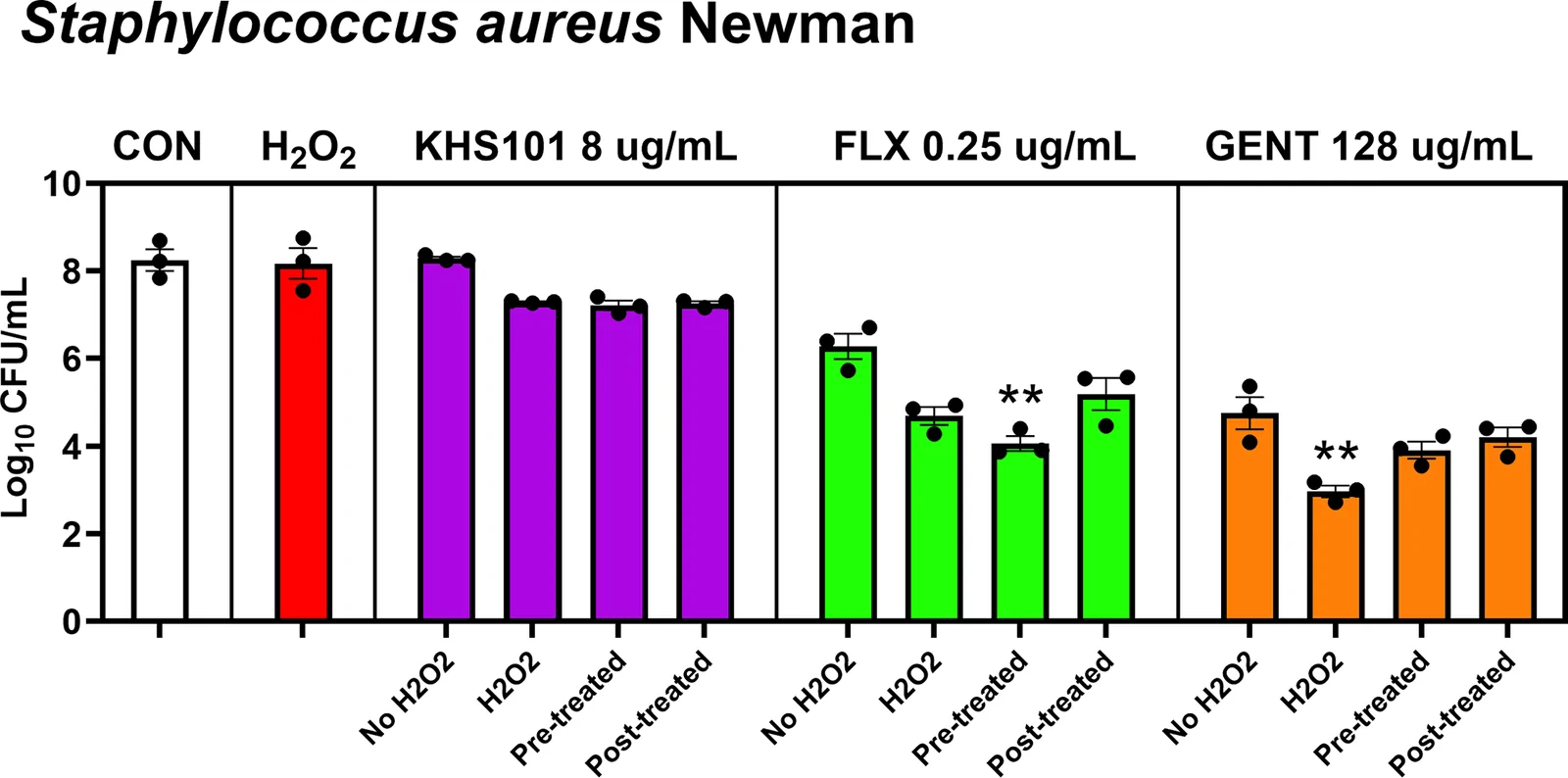
Cellular viability of the *S. aureus* Newman biofilms following treatment with KHS101 and H_2_O_2_. C.f.u. plate counts expressed as log_10_ c.f.u. ml^−1^ for KHS101, flucloxacillin (FLX) and gentamicin (GENT) in combination with 1 mM H_2_O_2_, either delivered together or pre- and post-treatment with the compound. Non-normally distributed data were analysed by Kruskal–Wallis with Dunn’s tests to determine the *P*-value for multiple comparisons across all variables within each treatment regime. * Indicates *P*<0.05, compared to UT control.

To further confirm the additive effects of H_2_O_2_, we performed plate count assay tests for one concentration of KHS101 or antibiotic, and each H_2_O_2_ combination. This was also done to determine if reduction in metabolic activity equated to cellular viability within the treated biofilms. The first concentration displaying metabolic activity above 50% when KHS101 or antibiotic was administered alone was selected: these are denoted in bold in Fig. 5. Due to lack of effect of treatments on *S. aureus* SH1000 metabolic activity, only the Newman strain was selected for plate counting experiments. Concentrations of KHS101 at 8 µg ml^−1^, flucloxacillin at 250 µg ml^−1^ and gentamicin at 128 µg ml^−1^ were tested. Evaluation of the resulting data in Fig. 6 for KHS101 showed no statistically significant reductions in viable cell counts compared to control or H_2_O_2_ alone. Flucloxacillin treatment showed a decrease in log_10_ c.f.u. ml^−1^ for all three groups of dual-treatments containing H_2_O_2_ compared to the antibiotic alone (no H_2_O_2_). The optimal effect can be observed for pre-treatment with H_2_O_2_, resulting in a 2-log_10_ difference to antibiotic alone, which significantly differed from UT controls following multiple comparisons (**P=0.0056). These results were largely in line with observations for the metabolic activity results shown in Fig. 5. In the case of gentamicin, all treatments reduced viable cell counts by >3.5 log_10_. A subtle improvement in antibiotic efficacy was observed when both therapies were delivered together simultaneously, compared to antibiotic alone (no H_2_O_2_; ~1 log_10_ reduction, **P=0.0041 when compared to UT controls). No significant differences were observed between treatments for any combinations within flucloxacillin or gentamicin groups. Although these results fail to support a case for H_2_O_2_–KHS101 interactions, they do document a potential role for biofilm sensitization with H_2_O_2_ either prior to or in combination with antibiotic treatment.

## Discussion

This study demonstrates that tolerance of *S. aureus* biofilms to CAP can be overcome through dual-therapy approaches without relying on direct antibiofilm activity from individual agents. Building on previous observations of biofilm-derived CAP tolerance, we identify here a repurposed small molecule called KHS101 that enhances plasma efficacy across mono-species and polymicrobial biofilms containing *S. aureus*.

Initial experiments conducted in this paper served to identify the most effective repurposed compound for further testing. This work built upon a previous publication from our group whereby a panel of over 1,000 compounds was initially screened as part of the Tocriscreen™ bioactive compound library. Results from that study identified five compounds that exhibited biofilm inhibition effects against several key fungal species including *C. albicans* [29]. Of those five identified, KHS101, darapladib and polygodial all showed some activity against *S. aureus*, with the former being the only compound that completely inhibited planktonic growth at any concentration tested. The idea of repurposing (or repositioning) compounds is not new, with multiple studies demonstrating that various libraries contain active agents that can combat bacterial pathogens including *S. aureus* [39–44]. Of note, the work of Van den Driessche *et al.* [44] screened over 700 compounds and identified Thioridazine, which, when combined with antibiotics such as flucloxacillin, was effective against MRSA biofilms [44]. From a fungal aspect, Abduljalil *et al.* [29] demonstrated that darapladib synergistically worked with Caspofungin in killing *C. albicans* biofilms [29]. However, there still remains a need to move away from antibiotic usage for clinical management of biofilm infections even if dual-therapy interventions can lower antibiotic concentrations needed. To account for this here, KHS101 treatment was combined with a unique six-jet CAP technology, which has previously been shown to work against a range of mono- and multi-species biofilm models but not all *S. aureus* strains [35].

Upon combining KHS101 with CAP, previously CAP-tolerant *S. aureus* Newman and SH1000 biofilms became susceptible to dual therapy, with Bliss and HSA analyses supporting enhanced combined antimicrobial activity beyond either monotherapy. This enhanced antimicrobial activity was an interesting observation, and thus the mechanism of action was further pursued, first using microscopic profiling. For this, the direct effects of KHS101 on the *S. aureus* biofilms were investigated, as this is the first reported case of its antibacterial activity. KHS101 has previously been investigated only for its neurogenic differentiation-inducing properties and in glioblastoma research [45, 46]. SEM imaging revealed KHS101 treatment induced morphological changes in the *S. aureus* cells, particularly in the Newman strain. This observed membrane blebbing is often accompanied by extracellular vesicle production as a form of bacterial defence to stress [47, 48]. Importantly, complementary TEM analysis of planktonic cells demonstrated that short-term KHS101 exposure did not result in overt ultrastructural damage, with cells retaining intact cell envelopes and showing no evidence of widespread lysis or cytoplasmic leakage. In parallel, SYTO-9/PI staining indicated subtle increases in PI-associated fluorescence following KHS101 exposure, suggesting limited membrane compromise rather than catastrophic membrane disruption. In human cells, KHS101 targets transforming acidic coiled-coil containing protein 3 (TACC3), which is important in cell mitosis and transcriptional functions, with elevated expression in various cancers [49]. Given that bacteria lack the TACC3 target of KHS101, it would be purely speculative to propose a mechanism by which this compound affects the *S. aureus* cells. What can be stated is that KHS101 treatment perturbs cellular structures but not lysis, changes that may promote CAP-mediated penetration and associated cellular death absent from monotherapy.

Evidence exists that CAP dual therapies can enhance efficacy of interventions, particularly antibiotics. Interestingly, the studies by Guo *et al.* [14, 50] reported that gas plasma and associated RONS can promote activity of various antibiotics in otherwise resistant MRSA strains, documenting a loss of ‘persister’ phenotype through sensitization of the biofilms both *in vitro* and *in vivo* [14, 50]. Similar to our previous observations, others have demonstrated that biofilm-embedded bacteria can exhibit ‘tolerance’ to direct plasma or plasma-activated liquids. To this end, Flynn *et al*. observed that *Acinetobacter baumannii* biofilms with denser biomass exhibited the greatest tolerance to plasma intervention [21], whilst Yang *et al.* [51] reported that plasma-activated saline only reduced *S. aureus* biofilm cell counts by 1-log_10_, compared to >5 log_10_ when treated alongside vancomycin [51]. However, these studies primarily report improved efficacy rather than explicitly addressing CAP tolerance as a biological phenomenon or investigating how it may be overcome. The novelty of the current study lies in its interrogation of CAP tolerance in biofilms and the demonstration that this tolerance can be broken through a rational dual-therapy approach using alternative novel therapies to antibiotics.

What still remains doubtful is the mechanism by which *S. aureus* is tolerant to CAP. Here, it was shown that bolus addition of H_2_O_2_ alone at concentrations produced by CAP was ineffective against *S. aureus* biofilms even for 24 h of treatment. This observation supports the involvement of additional CAP-derived reactive species, potentially including short-lived RONS. On this note, recent evidence has shown that different plasma-generated RONS are responsible for antimicrobial effects on various bacterial species. The studies from the laboratory of Mai-Prochnow have demonstrated that superoxide and ozone, two alternative oxidative radicals, were responsible for plasma-mediated antimicrobial effects against *Escherichia coli* [25, 52]. More recently, a study by Lim *et al.* [53] investigated plasma-derived RONS contribution to bacterial inactivation, highlighting that in fact H_2_O_2_ may not be the key driving compound in neutralizing bacteria, rather short-lived radicals such as peroxynitrite (ONOOH) were responsible for *S. aureus* killing. Additionally, they also reported that alternative RONS, such as hydroxyl radicals, were accountable for the death of *E. coli* cells, postulating that differences in cell wall thickness can impact RONS diffusion and associated damage [53]. However, such work was conducted on planktonic cells, failing to account for the biofilm phenotype and its protective barriers, including the extracellular matrix and associated polymers. Indeed, others have demonstrated that the biofilm matrix composition plays a critical role in modulating plasma susceptibility [54], with evidence that matrix components such as extracellular DNA can shield embedded cells, interfering with treatment efficacy [55]. Nevertheless, accumulating evidence indicates that plasma can exert a dual mode of action, whereby extracellular polymeric substances are directly modified or disrupted during treatment. For example, plasma exposure has been shown to physically etch biofilm biomass from surfaces and to remove extracellular polysaccharides from matrix components [56, 57].

An interesting observation from this work was the impact that strain heterogeneity can have on treatment efficacy. We previously demonstrated that different strains of *S. aureus* exhibited different levels of tolerance to CAP therapy, which drove the strain selection for the study presented here [35]. Although KHS101 used in tandem with CAP as a dual therapy broke the tolerance to the plasma, it is important to emphasize that this result may not extend to other *S. aureus* strains, including MRSA strains. This is particularly significant given that previous studies have shown that MRSA isolates are less susceptible to dielectric barrier discharge plasma therapy than methicillin-sensitive strains [58]. Our observed strain heterogeneity to treatment intervention was further evident when exploring the mode of action of KHS101 with H_2_O_2_, when compared to dual therapies with conventional antibiotics. For example, flucloxacillin and gentamicin treatment resulted in a decrease in metabolic activity at lower concentrations of each antibiotic when in combination with 1 mM H_2_O_2_ for *S. aureus* Newman, but no enhanced efficacy with the SH1000 strain. Although previous direct comparisons of *S. aureus* antibiotic susceptibility patterns are limited with these two strains, there is some published work that has shown that Newman is more resistant to gentamicin than SH1000 in direct comparison, albeit in planktonic forms [59]. While the mechanistic basis for this difference remains unclear, it is perhaps not unexpected given the distinct genetic backgrounds of the two strains. Indeed, comparative proteomic analyses have revealed significant differences in the expression of membrane and cell-wall-associated proteins, including adhesins, which can alter surface properties [60, 61]. Hypothetically, these cellular changes may influence interactions with external stressors (e.g. antibiotics and plasma-derived RONS). Future studies would benefit from transcriptomic profiling of *S. aureus* biofilms following CAP exposure to capture stress responses. This could involve targeted expression of cell wall markers, such as components of the VraSR two-component system, which are upregulated in response to stress signals [62]. Alternatively, a broader transcriptomic profiling following KHS101-CAP treatment would help distinguish the cause of this strain-dependent susceptibility. Indeed, such work would build upon RNA-sequencing-based approaches that have recently been reported for other organisms following CAP or plasma-derived water treatment [23, 63, 64].

An overarching theme emerging from these experiments is that certain interventions do not act primarily through direct antimicrobial activity (e.g. KHS101 or H_2_O_2_), but instead appear to sensitize biofilm-embedded cells to subsequent treatment. From a CAP standpoint, combining CAP with antibiotics has been shown to significantly enhance killing of *S. aureus* biofilms. It was proposed that such an effect was observed by CAP facilitating antibiotic penetration and increasing oxidative stress [14, 51]. Similarly, synergistic effects have been reported using dual therapies of H_2_O_2_ with conventional antibiotics, with the oxidative agent destabilizing the biofilm matrix, resulting in the formation of porous channels allowing for improved antibiotic entry points [65]. However, biofilm sensitization is unlikely to be limited to enhanced penetration alone. The work of Stewart *et al.* described how H_2_O_2_ can be actively neutralized within biofilms through enzymatic detoxification mechanisms (e.g. catalase production), resulting in steep concentration gradients that protect embedded cells from oxidative killing [66]. However, a ‘persister’ phenotype in biofilms cannot be directly captured from viability assays: cells may be viable but perturbed or stressed from initial treatments, ultimately lowering the threshold required for secondary antimicrobial insults to exert lethal effects. As shown in our microscopic findings here and previously [35], H_2_O_2_ or direct CAP therapy could be priming the *S. aureus* cells for follow-up antimicrobial treatments, even if monotherapies fail to kill.

An obvious limitation of the CAP-KHS101 vs H₂O₂-antibiotic/KHS101 assays in the present study is that they were performed using biofilms of different maturity, with the latter experiments conducted on 4 h models. As these biofilms represent an early, immature biofilm state and are likely to be less tolerant than mature communities, these findings should be interpreted as mechanistic evidence of potential sensitization under defined experimental conditions rather than as direct evidence of equivalent activity in mature biofilms. To this end, it has been reported that *S. aureus* biofilm age can influence antibiotic efficacy [67]. Future studies should further investigate this potential sensitization phenomenon against biofilms of varying maturity. A further translational consideration is the potential host-cell compatibility of KHS101. Although KHS101 has been investigated in mammalian systems and shown to induce cytotoxicity in glioblastoma models [45], the effects of the compound on wound-relevant host cells, such as keratinocytes and fibroblasts, remains unknown and fell outside the scope of this study. Future work should therefore assess mammalian cell viability at the concentrations and exposure durations required for CAP sensitization.

In summary, this study demonstrates that *S. aureus*-associated tolerance to CAP can be overcome through dual-therapy strategies consistent with biofilm sensitization rather than direct antimicrobial killing by KHS101 alone. The repurposed compound KHS101 emerged as a possible sensitizing candidate, rendering biofilms susceptible to subsequent CAP exposure, including in polymicrobial contexts. Mechanistic probing revealed that this effect could not be reproduced by H_2_O_2_ alone, highlighting the likely importance of other CAP-derived reactive species beyond H_2_O_2_. Collectively, these findings strengthen the ever-growing support for development of non-antibiotic adjuncts alongside other novel therapies such as plasma-derived technology.

## Supplementary material

10.1099/jmm.0.002193 Supplementary Material 1.

## Abbreviations

CAP: cold atmospheric plasma
CFE: colony-forming equivalent
EUCAST: European Committee for Antimicrobial Susceptibility Testing
HSA: highest single agent
LB: Luria–Bertani
MRSA: methicillin-resistant *Staphylococcus aureus*
PI: propidium iodide
PMA: propidium monoazide
pMIC: planktonic MIC
RONS: reactive oxygen and nitrogen species
SEM: scanning electron microscopy
TACC3: transforming acidic coiled-coil containing protein 3
TEM: transmission electron microscopy
UT: untreated
