# Supplementary material for: Biofilm sensitization to break Staphylococcus aureus tolerance to cold atmospheric plasma therapy
Source: J Med Microbiol. 2026 Aug 20;75(8):002193. doi: 10.1099/jmm.0.002193 (PMC13492704; doi:10.1099/jmm.0.002193)
Supplement: Supplementary file 1 [file jmm-75-02193-s001.pdf]

Biofilm Sensitisation to Break *Staphylococcus aureus* Tolerance to Cold Atmospheric Plasma Therapy

Abdullah Baz[1,2], Jontana Allkja[1,3,4], Muhanna Alshehri[1], Yao He[1], Zainab Bilal[1,6], Eve Hughes[1], Olivia Sealy[3], Craig Williams[5], Karen Faulds[6], Gordon Ramage[3], Jason L. Brown [1+]

Supplementary Table & Legend

Microbial species and strain	Source [reference]	Genetic background	Biofilm phenotype
***Staphylococcus aureus* Newman**	(Duthie and Lorenz, 1952)	*S. aureus* laboratory/clinical isolate widely used in virulence and biofilm studies	Reported as a relatively weak/low biofilm-forming strain in microtitre plates (Kean et al., 2017).
***Staphylococcus aureus* SH1000**	(Horsburgh et al., 2002)	Strain 8325-4 derivative with repaired rsbU and restored σB activity	High biofilm former in microtitre plates (Lauderdale et al., 2009, Lamret et al., 2021).
***Pseudomonas aeruginosa* PA14**	(Rahme et al., 1995)	Highly virulent clinical/reference strain	High biofilm former in microtitre plates (Kim and Park, 2013).
***Candida albicans* SC5314**	(Gillum et al., 1984)	Reference sequenced *C. albicans* strain	High biofilm former associated with yeast-hyphal morphogenesis (Ramage et al., 2001a, Ramage et al., 2001b)

Supplementary Table 1 – Summary of the microbial strains used throughout the study. *Staphylococcus aureus* Newman and SH1000 were included as genetically distinct *S. aureus* strains previously shown to exhibit varying levels of tolerance to CAP treatment. *Pseudomonas aeruginosa* PA14 and *Candida albicans* SC5314 were used in this study to create two triadic biofilm models with the *S. aureus* strains. Biofilm phenotypes are based on published reports using microtitre plates. Previous evidence has shown that these strains can form biofilms when grown alone or in combination within a cellulose matrix hydrogel model (Townsend et al., 2016, Baz et al., 2023)**.**

**Antimicrobial compound**	***Staphylococcus aureus* strain (PMIC)**	
	Newman	SH1000
Flucloxacillin	0.125-0.25 µg/mL	0.25-0.5 µg/mL
Gentamicin	2-4 µg/mL	2 µg/mL

Supplementary Table 2 – Planktonic minimum inhibitory concentrations of flucloxacillin and gentamicin against *Staphylococcus aureus*

Supplementary Figures & Legends

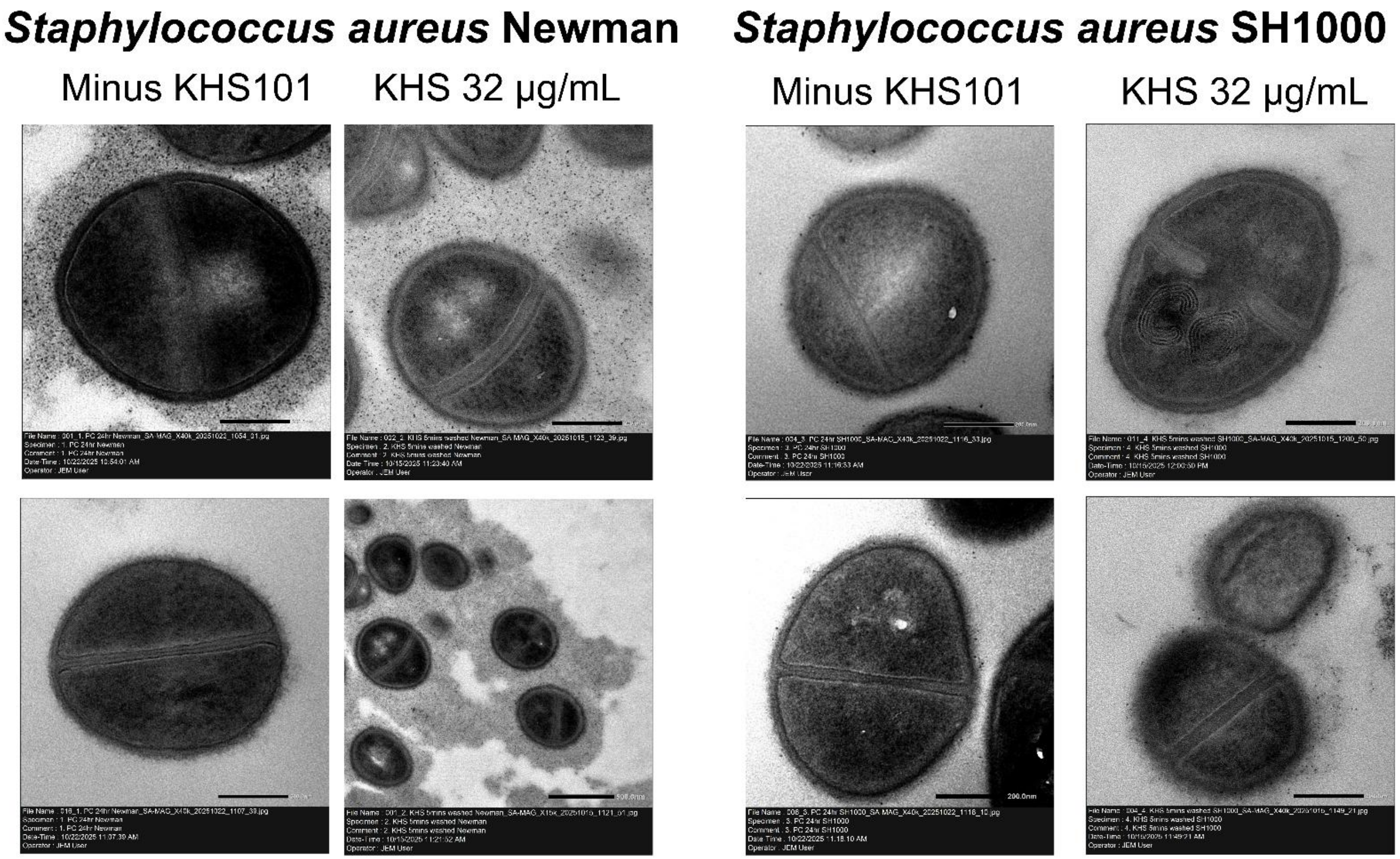

Supplementary Figure 1 – Transmission electron microscopic imaging of *Staphylococcus aureus* Newman and SH1000 treated with KHS101. Planktonic cells were treated for 5 minutes with 32 µg/mL KHS101 then visualized using a JEOL1200EX TEM. Untreated controls (minus KHS101) were imaged for comparative purposes. Images were taken at 40000x or 15000x magnification. Two representative images for both bacterial strains are included.

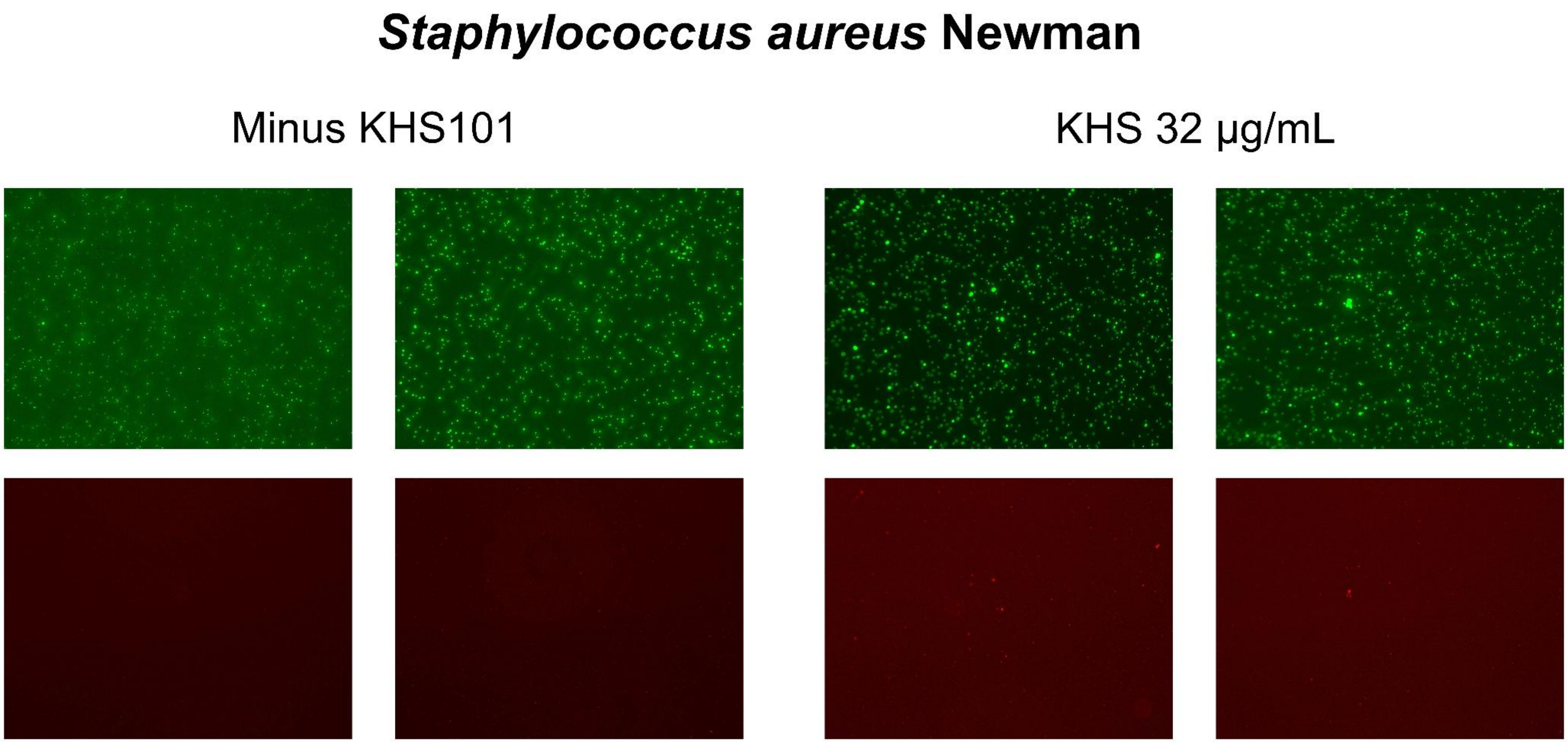

Supplementary Figure 2 – Representative fluorescence microscopy images of *S. aureus* Newman cells stained with SYTO-9 and propidium iodide following treatment with KHS101. *S. aureus* cells were treated with or without KHS101 at 32 µg/mL for 5 minutes. SYTO-9 staining is shown in green and indicates cells with intact membranes (top panels), while propidium iodide staining is shown in red and indicates membrane compromise (bottom panels). KHS101-treated cells showed a subtle increase in PI-associated red fluorescence compared with untreated controls, suggesting some but limited membrane permeability changes following short-term KHS101 exposure. Images taken using an EVOS M5000 imaging system at 10x magnification, with representative images from two fields of view from two biological replicates shown.

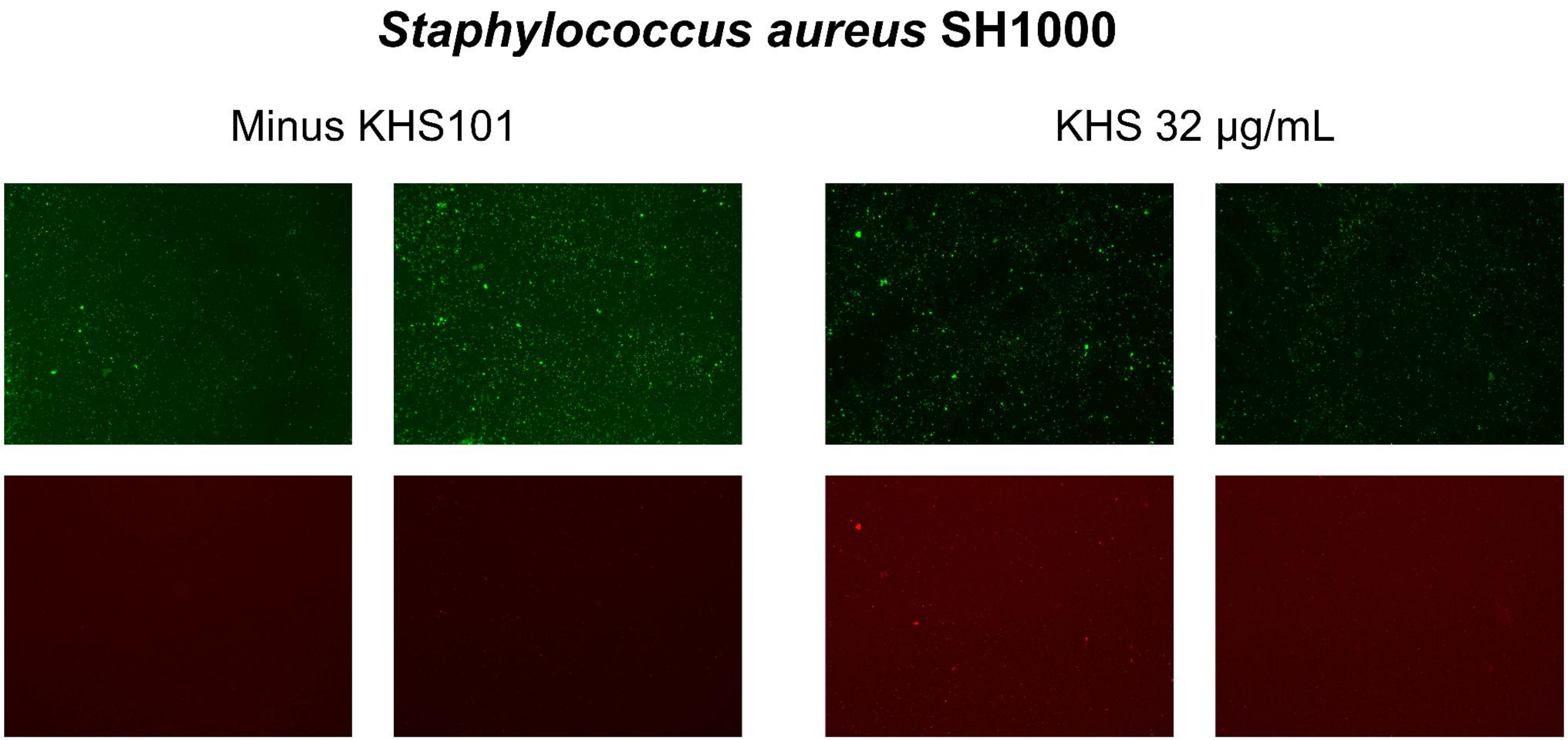

Supplementary Figure 3 – Representative fluorescence microscopy images of *S. aureus* SH1000 cells stained with SYTO-9 and propidium iodide following treatment with KHS101. *S. aureus* SH1000 were exposed to KHS101 at 32 µg/mL for 5 minutes prior to staining with SYTO-9 and propidium iodide. Green fluorescence corresponds to SYTO-9-positive cells (top panels), while red fluorescence corresponds to propidium iodide uptake associated with compromised membrane integrity (bottom panels). KHS101-treated cells displayed a modest increase in red fluorescence, consistent with partial membrane perturbation rather than widespread membrane disruption. Images captured at 10x magnification on the EVOS M5000 imaging system. Images are representative of two fields of view, from two independent biological replicates.

References

BAZ, A., BAKRI, A., BUTCHER, M., SHORT, B., GHIMIRE, B., GAUR, N., JENKINS, T., SHORT, R. D., RIGGIO, M., WILLIAMS, C., RAMAGE, G. & BROWN, J. L. 2023. Staphylococcus aureus strains exhibit heterogenous tolerance to direct cold atmospheric plasma therapy. *Biofilm,* 5**,** 100123.

DUTHIE, E. S. & LORENZ, L. L. 1952. Staphylococcal coagulase; mode of action and antigenicity. *J Gen Microbiol,* 6**,** 95-107.

GILLUM, A. M., TSAY, E. Y. & KIRSCH, D. R. 1984. Isolation of the Candida albicans gene for orotidine-5'-phosphate decarboxylase by complementation of S. cerevisiae ura3 and E. coli pyrF mutations. *Mol Gen Genet,* 198**,** 179-82.

HORSBURGH, M. J., AISH, J. L., WHITE, I. J., SHAW, L., LITHGOW, J. K. & FOSTER, S. J. 2002. sigmaB modulates virulence determinant expression and stress resistance: characterization of a functional rsbU strain derived from Staphylococcus aureus 8325-4. *J Bacteriol,* 184**,** 5457-67.

KEAN, R., RAJENDRAN, R., HAGGARTY, J., TOWNSEND, E. M., SHORT, B., BURGESS, K. E., LANG, S., MILLINGTON, O., MACKAY, W. G., WILLIAMS, C. & RAMAGE, G. 2017. Candida albicans Mycofilms Support Staphylococcus aureus Colonization and Enhances Miconazole Resistance in Dual-Species Interactions. *Front Microbiol,* 8**,** 258.

KIM, H. S. & PARK, H. D. 2013. Ginger extract inhibits biofilm formation by Pseudomonas aeruginosa PA14. *PLoS One,* 8**,** e76106.

LAMRET, F., VARIN-SIMON, J., VELARD, F., TERRYN, C., MONGARET, C., COLIN, M., GANGLOFF, S. C. & REFFUVEILLE, F. 2021. Staphylococcus aureus Strain-Dependent Biofilm Formation in Bone-Like Environment. *Front Microbiol,* 12**,** 714994.

LAUDERDALE, K. J., BOLES, B. R., CHEUNG, A. L. & HORSWILL, A. R. 2009. Interconnections between Sigma B, agr, and proteolytic activity in Staphylococcus aureus biofilm maturation. *Infect Immun,* 77**,** 1623-35.

RAHME, L. G., STEVENS, E. J., WOLFORT, S. F., SHAO, J., TOMPKINS, R. G. & AUSUBEL, F. M. 1995. Common virulence factors for bacterial pathogenicity in plants and animals. *Science,* 268**,** 1899-902.

RAMAGE, G., VANDE WALLE, K., WICKES, B. L. & LOPEZ-RIBOT, J. L. 2001a. Standardized method for in vitro antifungal susceptibility testing of Candida albicans biofilms. *Antimicrob Agents Chemother,* 45**,** 2475-9.

RAMAGE, G., VANDEWALLE, K., WICKES, B. L. & LOPEZ-RIBOT, J. L. 2001b. Characteristics of biofilm formation by Candida albicans. *Rev Iberoam Micol,* 18**,** 163-70.

TOWNSEND, E. M., SHERRY, L., RAJENDRAN, R., HANSOM, D., BUTCHER, J., MACKAY, W. G., WILLIAMS, C. & RAMAGE, G. 2016. Development and characterisation of a novel three-dimensional inter-kingdom wound biofilm model. *Biofouling,* 32**,** 1259-1270.
